# Combination of Stem Cells and Rehabilitation Therapies for Ischemic Stroke

**DOI:** 10.3390/biom11091316

**Published:** 2021-09-06

**Authors:** Reed Berlet, Stefan Anthony, Beverly Brooks, Zhen-Jie Wang, Nadia Sadanandan, Alex Shear, Blaise Cozene, Bella Gonzales-Portillo, Blake Parsons, Felipe Esparza Salazar, Alma R. Lezama Toledo, Germán Rivera Monroy, Joaquín Vega Gonzales-Portillo, Cesario V. Borlongan

**Affiliations:** 1 Chicago Medical School, Rosalind Franklin University of Medicine and Science, 3333 Green Bay Rd, North Chicago, IL 60064, USA; reed.berlet@my.rfums.org; 2 Lake Erie College of Osteopathic Medicine, 5000 Lakewood Ranch Boulevard, Bradenton, FL 34211, USA; santhony01304@med.lecom.edu; 3 Department of Neurosurgery and Brain Repair, Morsani College of Medicine, University of South Florida, 12901 Bruce B Downs Blvd, Tampa, FL 33612, USA; beverly14@usf.edu (B.B.); zwang94@usf.edu (Z.-J.W.); 4 Georgetown University, 3700 O St NW, Washington, DC 20057, USA; nas146@georgetown.edu; 5 University of Florida, 205 Fletcher Drive, Gainesville, FL 32611, USA; a.shear@ufl.edu; 6 Tulane University, 6823 St. Charles Ave, New Orleans, LA 70118, USA; bcozene@tulane.edu; 7 Northwestern University, 633 Clark St, Evanston, IL 60208, USA; bellagonzales-portillo2024@u.northwestern.edu; 8 Washington and Lee University, 204 W Washington St, Lexington, VA 24450, USA; parsonsb24@mail.wlu.edu; 9 Centro de Investigación en Ciencias de la Salud (CICSA), FCS, Universidad Anáhuac México Campus Norte, Huixquilucan 52786, Mexico; felipe.esparzas69@anahuac.mx (F.E.S.); alma.lezamato@anahuac.mx (A.R.L.T.); german.riveramo@anahuac.mx (G.R.M.); 10 Universidad Peruana de Ciencias Aplicadas, Prolongación Primavera 2390, Lima 15023, Peru; jovgagp95@gmail.com; 11 Center of Excellence for Aging and Brain Repair, Morsani College of Medicine, University of South Florida, 12901 Bruce B Downs Blvd, Tampa, FL 33612, USA

**Keywords:** stroke, stem cell therapy, bone marrow-derived mesenchymal stem cells, neural stem cells, endothelial progenitor cells, neuroinflammation, rehabilitation therapy

## Abstract

Stem cell transplantation with rehabilitation therapy presents an effective stroke treatment. Here, we discuss current breakthroughs in stem cell research along with rehabilitation strategies that may have a synergistic outcome when combined together after stroke. Indeed, stem cell transplantation offers a promising new approach and may add to current rehabilitation therapies. By reviewing the pathophysiology of stroke and the mechanisms by which stem cells and rehabilitation attenuate this inflammatory process, we hypothesize that a combined therapy will provide better functional outcomes for patients. Using current preclinical data, we explore the prominent types of stem cells, the existing theories for stem cell repair, rehabilitation treatments inside the brain, rehabilitation modalities outside the brain, and evidence pertaining to the benefits of combined therapy. In this review article, we assess the advantages and disadvantages of using stem cell transplantation with rehabilitation to mitigate the devastating effects of stroke.

## 1. Introduction 

Advances in medical accessibility, technology, and treatment over the past century have increased the average human life expectancy, but this comes with higher risks for neurodegenerative diseases and disorders [1,2]. The economic and social costs of neurological diseases, such as stroke, cause significant distress for patients, families, and society as a whole [3]. As of yet, primary treatments focus mainly on managing the progression of the disease and treating symptoms rather than curing the underlying causes of many neurological conditions. The current mainstay of treatment for ischemic stroke is thrombolytic reperfusion through tissue plasminogen activator (tPA) infusion, but the narrow therapeutic window and associated adverse effects have not eliminated stroke as a major cause of mortality and morbidity around the world [4]. Other therapies such as endovascular mechanical thrombectomy have a wider therapeutic window, but the window is still hours rather than days, and the damage from prolonged brain ischemia is often irreversible [5,6]. Preventative measures such as anticoagulant therapy for known causes of stroke, like atrial fibrillation, are far from universal and increase the risk of hemorrhage [7]. Physical therapy, speech therapy, and occupational therapy rehabilitation for stroke patients over a longer period may help recover some function, but patients are commonly left disabled [8]. The importance of developing novel therapies with greater therapeutic windows for stroke cannot be overstated, and the investigation of regenerative rather than symptomatic treatment for neurological disorders is paramount to moving forward with new therapies.

Stem cells represent an exciting potential therapy for ischemic stroke that may extend the therapeutic window of stroke treatment. Stem cell treatment demonstrates apparent beneficial effects in preclinical stroke models by reducing infarct size and improving behavioral and histological deficits [9,10,11]. Furthermore, preclinical experimentation has established that stem cell therapies are effective days after the ischemic stroke event, allowing for an extension of the therapeutic window for stroke treatment [11,12]. Despite the potential of stem cell therapy established in preclinical stroke models, there exists a frustrating disconnect between results when translated to clinical experimentation [9,13]. Even with promising data from small, prospective, and phase I clinical studies, data from randomized clinical trials have remained equivocal for supporting stem cell therapy in stroke patients [9,14,15]. Some clinical investigations have demonstrated modest therapeutic effects of stem cell therapy in stroke, with several showing safety but no efficacy [16]. Research must emphasize determining the optimal clinical stem cell route of administration, dosage, and timing to maximize the therapeutic potential of stem cells. 

Post-ischemic brain tissue is characterized by a pro-inflammatory environment that prevents stem cells from establishing and inducing neuroregeneration [13]. Thus, combinate administration with different biomaterials and drugs has been analyzed with the aim of improving stem cell transplantation [12,13]. Biomaterials have gained particular interest due to the great variety of molecular compositions and their roles in recovery [12]. Biomaterials act as scaffolds that mechanically protect stem cells during their migration to the site of action [17,18]. Similar to biomaterials, the use of drugs in combination with stem cell transplantation is being investigated as a potential booster for these therapeutic benefits [13,19]. Concomitant administration with drugs and the usage of biomaterials stand as promising adjuvants for stem cell therapies in stroke [20,21]. Nevertheless, more data is needed to determine the best compounds, correct dosage, therapeutic window, and specific patient characteristics required for this treatment to have major success [13,22]. As the number of clinical trials testing stem cell transplantation increases each year [9], science faces the challenge of public concern and ethical controversy [23,24]. Researchers must mitigate public fear and misconception of this therapy by using evidence-based medicine and thorough application of preclinical studies.

Stem cell therapy has emerged as a potential stroke treatment to repair and regenerate the brain via exogenous stem cell transplantation or stimulating endogenous stem cells [25]. Stoke entails primary and secondary cell death pathways that create a harsh microenvironment [26]. This non-conductive microenvironment limits the survival and growth of exogenous and endogenous stem cells, limiting brain repair [12,27]. Strategies designed to enhance the regenerative features of the microenvironment may promote improved brain repair.

Combining these effective therapies thoughtfully for stroke rehabilitation may be the future of stroke treatment and functional recovery. The subsequent sections will focus on the use of stem cells and biomaterials for rehabilitation of the stroke brain.

### 1.1. Current Standing of Stem Cells as a Stand-Alone Therapeutic for Stroke 

The rise of stem cell therapy for stroke is critical for the advancement of stroke treatment, especially for post-stroke rehabilitation and long-term patient recovery. The only current FDA-approved drug for acute stroke therapy, tPA, is limited due to its short therapeutic window and risk of hemorrhage. This highlights the need for further research into an alternative stroke treatment. Remarkably, stem cell transplantation has exhibited promising outcomes due to its capacity for self-renewal, differentiation into tissue-specific cells, and modulation of endogenous neurogenesis [28]. Furthermore, preclinical studies have elucidated the regenerative effects of stem cell therapy, including neurological rehabilitation and structural recovery [28]. These findings shed light on the growing therapeutic potential of stem cell transplantation in stroke patients. Overall, exogenous stem cell therapy culminates in the regeneration of injured cells, neuroprotection, and recruitment of endogenous stem cells, all of which aid in alleviating stroke-induced damage and promoting long-term rehabilitation. 

### 1.2. Exogenous Stem Cell Therapy for Stroke

#### 1.2.1. Novel Preclinical Evidence

Over the last decade, experimental stroke models have demonstrated the efficacy of transplanted stem cells with respect to their survival, functional integration, and positive impact on neurological and motor improvement [9]. Various stem cell lines have been utilized in animal models of stroke, resulting in behavioral recovery and positive histologic effects. Stem cell types examined in preclinical stroke models include embryonic stem cells, hematopoietic stem cells (HSCs), neural stem cells (NSCs), mesenchymal stem cells (MSCs), adult tissue-derived stem cells, and induced pluripotent stem cells (IPSCs) [28,29]. Preclinical studies for stem cell transplantation post-stroke, particularly MSCs and NSCs, have shown improved cognitive and motor function through replacement, neuroprotection, growth factor secretion (bystander effect), neurotrophic factor secretion, extracellular matrix (ECM) remodeling, recruitment of endogenous NSCs (biobridge), immune regulation, neurogenesis, angiogenesis, astrogenesis, synaptogenesis, and oligodendrogenesis [12,13,30,31,32,33,34,35,36]. To determine the optimal cell type to use for exogenous stem cell transplantation, issues surrounding efficacy, availability, and ethics are to be considered [29].

The powerful analytical tools of preclinical data offer an objective review of the efficacy of using stem cells as a treatment to improve stroke outcomes. In addition, a deeper understanding of the mechanism underlying the therapeutic actions of stem cells is warranted before clinical application [37]. Notably, the application of stem cells in preclinical stroke models has resulted in a significantly improved neurological function score and infarct volume [38]. Of note, MSCs, NSCs, and HSCs used for regenerative therapy of stroke can protect against tissue damage, promote injured tissue repair, and increase functional recovery [39,40]. Stem cells provide neuroprotection and restorative properties that ameliorate the loss of astrocytes, neurons, blood vessels, and oligodendrocytes, making their therapeutic potential quite promising [19]. Multiple preclinical studies have elaborated how stem cells afford functional benefits in animal stroke models. Isolated stem cells from menstrual blood treated with oxygen-glucose deprivation display neuronal phenotypic markers, such as Nestin and microtubule-associated protein 2 (MAP2) and significantly increase sensorimotor improvement after transplanted into stroke-induced rats [41]. Ntera2 cells transplanted into stroke lesioned rats show the ability of these cells to secrete glial cell line-derived neurotrophic factor (GDNF) and lead to an improvement in sensorimotor function via neuronal rescue [42]. Increases in Ki67, a marker for cell proliferation, and MAP2, a neuronal marker, are observed after amniotic fluid-derived stem cells show differentiation into NSCs, leading to increased sensorimotor improvement [43]. CD133+ cells derived from human bone marrow also exhibit sensorimotor benefits in stroke lesioned rats [44].

#### 1.2.2. Various Exogenous Stem Cell Types Exhibit the Capacity to Recruit Endogenous Stem Cells 

Notably, stem cell therapy demonstrates the capacity to bolster innate repair mechanisms following ischemic stroke via endogenous stem cell recruitment. Exogenous stem cell transplantation can promote neurogenesis and angiogenesis and also recruit progenitor cells of the neurogenic niches to the ischemic site [36,45,46]. In addition, transplanted stem cells secrete paracrine factors that target the neurogenic niches, increasing the proliferation, differentiation, and migration of progenitor cells. Endogenous stem cell recruitment by exogenous stem cells has recently been investigated with MSCs, NSCs, endothelial progenitor cells (EPCs), adipose stem cells, and urine-derived stem cells (USC-Exos).

To direct progenitor cell homing to the injured site, exogenous stem cells construct a biobridge from the neurogenic niches to the ischemic region [47,48]. Following ischemic stroke, injured neurons release inflammatory factors, which stimulate exogenous stem cells to upregulate neurogenesis in the neurogenic niches and direct the newly proliferated neural progenitor cells (NPCs) to migrate towards the ischemic region via the corpus callosum [36,45,49]. Interestingly, transplanted MSC concentration in rats 100 days post-traumatic brain injury (TBI) was low despite the presence of NSCs, highlighting a replacement of transplanted MSCs with endogenous NSCs that migrated from the neurogenic niches of the rat brain to the infarct area (the biobridge) [11,50,51]. Transplanted stem cells can utilize matrix metalloproteinases (MMPs) as a chemoattractant to guide endogenous progenitors from the subventricular zone (SVZ) to the injured site [47,48]. Migrating NSCs may evoke adhesion molecules such as integrin β-1 to create a biobridge from the transplanted stem cells [47,48]. The biobridge contains enhanced levels of MMP-9 that advance ECM remodeling to facilitate a pathway for endogenous cells to travel [45,52]. These endogenous NSCs replace the transplanted cells, continue the anti-inflammatory cascade, and differentiate into tissue-specific neurons, astrocytes, and oligodendrocytes [12,53,54]. Transplanted NSCs in stroke lesioned rats show neurological and behavioral recovery, highlighting the endogenous cells’ migratory importance [45,52]. Altogether, exogenous stem cell-induced neurogenesis and biobridge formation stand as additional mechanisms underlying the efficacy of stem cell therapy for stroke. These processes should be further investigated to advance treatment for post-stroke rehabilitation. 

##### MSCs

Exogenous MSC transplantation provides a conducive environment that promotes tissue repair by secreting a diverse array of bioactive molecules known collectively as secretome [55]. These biomolecules include growth factors, cytokines, chemokines, neurotrophins, and extracellular vesicles [55]. Thus, MSC transplantation suppresses inflammation, reduces cell death, promotes angiogenesis, and stimulates neurogenesis [55]. MSCs lower overall inflammation, ameliorate potentially toxic environments, and increase neurotrophic factor release, enabling both endogenous NSC survival and function [56]. Therefore, they enhance cell proliferation, migration, and differentiation into functional mature cells. Moreover, MSC administration as stroke therapy culminates in ameliorating the infarct and penumbra, sometimes leading to complete functional recovery in animal models [56]. In vivo, intravenous (IV) infusion of MSCs leads to alleviated behavioral deficits, an increase in regional cerebral blood flow, and rehabilitation of cerebral microvasculature [57]. Altogether, these preclinical findings support the use of MSCs in clinical trials for stroke therapy. 

Novel preclinical studies have illustrated the potential of MSCs to boost intrinsic repair processes following ischemic stroke via endogenous stem cell recruitment [58,59,60,61]. Treatment with MSC-derived exosomes may improve synaptic plasticity, angiogenesis, axon and myelin density, and neuroblast migration to the ischemic region [59,62,63]. MSC-derived exosomes promote axonal sprouting from healthy cortical tissue to damaged striatal tissue, thereby upregulating axon levels in the striatum [59,60]. Treatment with these exosomes results in a higher count of oligodendrocyte progenitor cells (OPCs), mature oligodendrocytes, and myelinated axons [59,60], alluding to their effects on endogenous stem cells [64]. Furthermore, MSC-derived exosomes can strengthen endogenous repair mechanisms that specifically target white matter injury [65]. Additionally, bone marrow-derived mesenchymal stem cell (BM-MSCs) administration results in a heightened division of endogenous NPCs in the SVZ [61,66,67,68]. BM-MSCs mitigate stroke-induced neurological deficits by decreasing the infarct size and fostering angiogenesis, synaptogenesis, and neurogenesis towards the infarction [61,69]. Thus, exogenous MSCs recruit newly proliferated neural precursors to the injured site, culminating in the restoration of neuronal loss. 

BM-MSCs also have relevant effects on astrocytes and microglia. Glial cells, such as astrocytes, have many roles in brain repair and damage after stroke. In the acute phase, the glial cells are oriented to a pro-inflammatory phenotype, expediting phagocytosis and inflammation through molecular mediators like cytokines and chemokines and recruiting immune cells [70]. However, they also have the ability to transform into an anti-inflammatory phenotype and engage in functions such as maintenance of the neurovascular unit (NVU), neuroprotection, immunomodulation, antioxidation, and modulation of synaptic function [71]. Transplanted MSCs stimulate astrocytes to release anti-inflammatory factors, repair-focused neurotrophic factors, and growth factors such as insulin-like growth factor-1 (IGF-1), vascular endothelial growth factor (VEGF), epidermal growth factor (EGF), and basic fibroblast growth factor (bFGF) [72]. MSCs enhance bone morphogenic proteins (BMPs) 2/4 expression in ischemic astrocytes, leading to increased gliogenesis in the SVZ and improved functional recovery [71,73].

Moreover, MSCs derived from adipose tissue demonstrate the potential to promote neurogenesis and post-stroke rehabilitation [66,74]. Transplantation of adipose tissue-derived MSC sheets in middle cerebral artery occlusion (MCAO) rats results in a considerable boost in angiogenesis and neurogenesis, suggesting their therapeutic potential in human patients [75]. Overall, MSCs ameliorate the infarct region and reduce cell death in the penumbra, in part by promoting endogenous regenerative methods.

MSCs use inflammatory-mediated signaling pathways to recruit endogenous cells that migrate via a biobridge mechanism. Stromal cell-derived factor-1 (SDF-1) receptors exist in infarct areas and lure chemokine receptor type 4 (CXC4) while activating MMP-9 [76,77]. With enhanced MMP-9 levels, MSCs harness the ability to remodel the ECM to form a biobridge that aids in activating endogenous cells towards infarct regions [50]. The endogenous NSCs replace the transplanted MSCs [50]. Additionally, MSCs release elevated levels of immunomodulatory mediators such as IL-6 and prostaglandin E2 (PGE2) [78,79], which induce angiogenesis that is necessary for endogenous repair [80,81]. Besides the increased secretion of MMP-9, MSCs release fibroblast growth factor 2 (FGF2) [82,83]. The secretion of bFGF may affect VEGF and VEGF receptor 2 (VEGFR2) expression on endogenous cells, leading to angiogenesis and enhanced proliferation of endothelial cells [84]. Therefore, through various inflammatory and neurotrophic factors, MSCs can recruit endothelial cells and initiate blood flow restoration in the ischemic area, promoting post-stroke rehabilitation. Moreover, MSCs may secrete brain-derived neurotrophic factor (BDNF), which represents a neurotrophin of therapeutic interest. BDNF stimulates neuronal survival and differentiation that can attenuate infarct regions, increase repair, and further neurogenesis [85,86]. MSCs also recruit inflammatory cells to aid in decreasing the infarct volume [87,88]. In short, exogenous MSCs exhibit the capacity to recruit a wide variety of cell types, such as NPCs, endothelial cells, and immune cells to regenerate neuronal loss in the infarct region and protect living tissue from further stroke-induced injury. These therapeutic actions of MSCs accentuate the robust efficacy of these cells for the treatment of post-stroke impairment. 

MSC clinical trials have had variable results [33]. The safety and efficacy of MSC transplantation evaluated in a long-term follow-up aimed to observe the long-term side effects [89]. Robust functional improvement was found in patients undergoing the therapy [89]. Similarly, autologous modified MSC administration was safe and effective in chronic major stroke patients [90]. Compared to the control group, patients with MSC treatment demonstrated substantial mitigation of lower extremity motor impairments [90]. Of note, phase I/II clinical trials conducted by Bang et al. showed an increase in Barthel’s index 90 days post-stroke when given IV BM-MSCs to patients [91]. Infused IV bone marrow-derived MSC in a clinical trial shows an increase in both the Fugl-Meyer index and modified Barthel index [92]. Despite some clinical studies demonstrating moderate efficacy, there exists a frustrating disconnect between preclinical and clinical results [9,13]. This incongruity is likely due to multiple factors such as: suboptimal dosage, lack of use of clinical-grade cell lines, and timing of stem cell administration [9,16,93]. With considerable evidence demonstrating clinical safety of MSCs in stroke patients [9,31,90], future clinical research should further evaluate the efficacy of MSCs as a stroke therapy and examine how stem cell therapeutic mechanisms can be better utilized to see greater preclinical translation to clinical investigations. 

##### NSCs

NSC transplants are advantageous in the restoration of brain structure and function after injury. They offer beneficial characteristics, including high tissue-specific regeneration potential, due to their ability to proliferate and differentiate into different cell types of the corresponding microenvironment [48]. Other advantages of NSCs include easier delivery compared to other stem cell types (e.g., MSCs) and their low rejection rate [48]. NSC transplantation also displays notable chemotaxis and migration abilities to the damaged area [48]. Furthermore, CTX0E03 is the most promising NSCs line, with the ability to enhance behavioral recovery in stroke patients [48]. Altogether, exogenous NSC treatment shows robust therapeutic efficacy for stroke treatment because of its high proliferative, differentiative, and migratory capacity. 

In Parkinson’s disease (PD), human neural stem cells (hNSCs) stimulate endogenous repair mechanisms in the SVZ, as indicated by upregulated neurotrophic and anti-inflammatory factors in the neurogenic region, which correlates with alleviated motor and neurological impairments [30,94,95]. Transplanted NSCs in stroke patients may recapitulate the repair mechanism seen in PD, presenting NSCs as a promising therapeutic strategy to use for cell recruitment. Notably, transplanted human embryonic NSCs significantly upregulated host cell proliferation in the SVZ for 14 days in MCAO rats [96]. Similarly, human NSC delivery into aged rats with ischemic stroke results in heightened endogenous NPC proliferation in the subgranular zone (SGZ) [97]. Likewise, NSC treatment significantly upregulates neurogenesis and angiogenesis in young and aged rats with ischemic stroke [12,98,99].

In addition to stimulating neurogenesis, transplanted NSCs may recruit endogenous progenitor cells by helping them migrate to the ischemic site. An NSC-infused polymer scaffold insertion into the brains of mice exposed to ischemia results in a substantial increase in neurite growth from both exogenous NSC and host cell-derived neurons [100]. Interestingly, transplanted NSCs guided neurite development, leading to the restoration of damaged cortical tissue and ameliorated neuroinflammation [100]. The interplay between NSCs and host cells may be fostered by biobridges, allowing for increased host cell differentiation and coordinated axonal outgrowth to specific targets [100]. Notably, the immortalized NSC line, CTX0E03, substantially upregulates neuroblast proliferation in the striatum and recruits microglia, which correlates with sensorimotor improvement [101]. Therefore, transplanted NSCs not only recruit neuroblasts but also stimulate microglia proliferation, indicating an anti-inflammatory mechanism. In neonatal hypoxia-ischemia mice, NPC transplantation leads to the recruitment of endogenous oligodendrocytes to the corpus callosum, potentially via the secretion of paracrine factors and the formation of a biobridge [102]. Increased migration of endogenous oligodendrocytes to the injured site results in significant re-myelination, which is correlated with alleviated motor deficits [102]. Human NSCs transplanted intracerebrally were administered to 13 stroke patients and led to an improved National Institutes of Health Stroke Scale (NIHSS) after two years [103]. All in all, NSCs exhibit the capacity to recruit NPCs, as well as other glial cells, such as microglia and oligodendrocytes, to the injured site.

##### Other Cell Types 

In addition to MSCs and NSCs, EPCs, adipose stem cells, and USC-Exos can also target intrinsic repair mechanisms. EPCs demonstrate the potential to effectively rehabilitate the impaired neurovascular networks post-ischemia by promoting angiogenesis and neurogenesis [104] and ameliorating blood–brain barrier (BBB) permeability [105]. EPCs derived from bone marrow exhibit the ability to mitigate stroke-induced endothelial cell impairment, thereby alleviating inflammation and BBB permeability [105]. Notably, the administration of human umbilical cord blood-derived EPCs in MCAO rats led to an increase in endogenous cell proliferation in the SVZ and dentate gyrus and heightens neurogenesis and angiogenesis [106]. Furthermore, exogenous EPCs can recruit neural precursors in the neurogenic niches, as evidenced by the increased neurogenesis following EPC transplantation. EPCs contribute to angiogenesis after stroke, potentially through the secretion of VEGF, SDF-1, and platelet-derived growth factor (PDGF) [104,107]. Altogether, transplanted EPCs may foster endogenous stem cell recruitment and subsequent rehabilitation via neurogenesis and angiogenesis. However, the capacity of EPCs to help endogenous stem cells migrate from neurogenic niches to the injured region needs to be further explored.

Additionally, the use of exogenous adipose stem cells in conjunction with a hyaluronic acid biomaterial scaffold in stroke mice leads to a significant increase in neuroblast, glial cell, and endothelial cell proliferation in the SVZ. The treatment also directs the migration of newly proliferated cells to the injured site [108]. Therefore, exogenous adipose stem cells can recruit a variety of cell types to the ischemic region.

With respect to USC-Exos, when these exosomes are introduced to an NSC culture exposed to oxygen-glucose deprivation (OGD/R), NSC proliferation and differentiation significantly increases [66]. In vivo, USC-Exos upregulates neurogenesis, thereby engendering functional rehabilitation post-stroke [66]. Furthermore, USC-Exos demonstrates the ability to recruit NPCs via the promotion of endogenous neurogenesis.

The mechanism behind adipose stem cells and USC-induced recruitment of endogenous stem cells in stroke warrants further investigation. EPCs, adipose stem cells, and USC-Exos should be examined more extensively in preclinical setting for stroke treatment, particularly regarding their ability to capitalize on endogenous repair processes.

#### 1.2.3. Stem Cell Source, Administration Route, Dose, and Timing for Stroke Treatment

Given the potentially high therapeutic value of stem cell therapy for stroke [31], further investigation is needed to optimize stem cell dosage, timing, and administration route, as well as to identify the best stem cell source for therapies. Ethical and logistical concerns with fetal and embryonic stem cells have made their use challenging for brain disorders [9,13]. Thus, bone marrow-derived stem cells have been the focus of preclinical and clinical studies for stroke due to their well-established safety and adult tissue origin while still retaining stem cell phenotypic characteristics [13,16]. Several stem cell subsets have been developed from bone marrow, such as MSCs, EPCs, SB623, multipotent adult progenitor cells, and multilineage-differentiation stress-enduring cells along with many others [9,13]. While there were previous concerns regarding the safety of stem cell therapy due to the potential for tumorigenicity, there exists strong evidence for the safety of MSC transplantation as stroke therapy [9,31,90]. Regarding stem cell sources, not only does the age of the recipient influence the efficacy of stem cell transplantation but so does the age of the stem cell donor, since aging results in a decline of function in both exogenous and endogenous stem cells [31,109,110]. Clinical stem cell therapy may also require immunosuppression, which can be problematic for patients. Ideally, selected stem cell sources should not be immunogenic or be engineered to be hypoimmunogenic [31,111]. 

There are multiple routes of administration for stem cell stroke therapy; however, IV, intracerebral (IC), and intra-arterial (IA) are the most well studied. IV administration is minimally invasive but limited by less brain tissue penetration and more widespread organ stem cell accumulation [11,12]. While IA offers greater brain tissue penetration than IV, it bears a significant clotting risk [11,12]. IC provides a high therapeutic value through greater brain tissue penetration but is a highly invasive procedure with risks for many adverse effects, such as seizures [11,12]. Intranasal administration is a newer minimally invasive administration route for stem cells with a potentially high therapeutic value [12,13]. If intranasal administration has an equal or greater therapeutic effect to the other transplantation routes, it will likely be the most ideal and practical clinical cell therapy administration route. Nonetheless, the optimal clinical stem cell route of transplantation requires further investigation. 

Determining appropriate therapeutic indices for stem cell transplantation is vital as insufficient dosages will not produce therapeutic effects while larger dosages carry greater risks for adverse effects, such as tumorigenesis or clot formation [11,31]. Current preclinical findings indicate that IV doses between 1 × 10^6^ to 5 × 10^6^ MSCs/kg body weight permit significant therapeutic improvements in stroke animal models [12,112,113]. Despite this, effective stem cell dosage is highly dependent on the host’s brain microenvironment and is likely variable [12,27]. Both dosage and timing for stem cell therapy need further optimization. Administration of cell therapy within the seven-day post-brain injury time frame has been suggested [12,27]; however, there are indications that a narrower clinical window within a few days of stroke onset may be the most optimal [9,11,16]. Despite the specific optimal timing of transplantation being unknown, it will likely fall within the one-seven day post-stroke time frame, representing a significant extension of the stroke therapeutic window when compared to traditional thrombolytic therapeutics. 

#### 1.2.4. Stems Cells and Stroke in the Aged Brain

Old age is associated with greater susceptibility to stroke and poor recovery from brain injury [114]. The aged brain represents a primary concern in formulating stroke therapies as a majority of stroke patients are over the age of 65 and half of all strokes occur in patients over the age of 75 [115]. The SVZ represents the neurogenic niche with the largest source of stem cells [116]. While NSCs remain present in the brain throughout adult life, during aging the germinal potential of neurogenic niches like the SVZ declines [116]. The aged brain undergoes accelerated progression of the ischemic area and delayed neurological recovery. This is due to greater BBB permeability, decreased antioxidant capacity, greater axonal sprouting, and inflammation in the aged brain [117]. Age-related activation of microglia in response to ischemia is a major mechanism by which greater neuroinflammation worsens stroke outcomes in the aged brain [117]. While the restorative potential of the brain exists during senescence, the decreased proliferation of endogenous NPCs limits neurological recovery [115]. The various mechanisms underlying this decline are not fully understood, however, aging likely limits SVZ neurogenic potential by disrupting its cellular organization [118]. Of note, despite reduced potential of NSC regeneration, the aged brain appears to preserve the regeneration of oligodendrocytes [116]. Overall, there exists a clear limitation in the neurogenic capacity of the aged brain, thus studies working to see the translation of stem cell therapies and other regenerative therapies to the clinic must account for the limited neurogenic potential of the aged brain in preclinical studies.

Fortunately, neurogenic niches can be enhanced by several therapies such as stem cell therapy, physical activity, enriched environment, or pharmaceuticals [118,119,120]. Furthermore, electrical stimulation therapies in post-stroke aged rats facilitated a functional improvement of spatial long-term memory and generated a significant increase in the number of newly born doublecortin cells in the neurogenic niches of the infarcted brain hemisphere [118]. Utilization of iPSCs allows for enhanced neurogenesis via iPSC differentiation into functional neurons, ameliorating deficits in the stroke-injured aged brain [121]. The decreased neuroregenerative environment of the aged brain does not prevent the effects of stem cells on regenerative brain remodeling, endogenous neurogenesis, or functional neurologic recovery [115]. Stem cell based therapies hold promise as an ischemic stroke therapy, especially if enhanced by additional therapies; however, the failure of clinical trials is partly due to the limited consideration of the aged brain. Future research must emphasize the pathological mechanisms present in the aged brain and how to target these mechanisms to fully employ the potential of stem cell based therapies. 

#### 1.2.5. Neuroprotective Effects of Exogenous Stem Cells 

Exogenous stem cell transplants bear neuroprotective properties against the detrimental effects of ischemic stroke in the brain via the replacement of damaged tissue, stimulation of angiogenesis or neurogenesis, anti-inflammatory effects, and antiapoptotic properties [29,122,123,124,125,126,127,128,129,130]. The most direct mechanism by which neurological restoration occurs is by the replacement of damaged brain tissue with neural cells differentiated from stem cells [131,132,133]. 

In the ischemic penumbra, the microenvironment is hostile and contains neurons that can be rescued if targeted appropriately [31,33]. Innate qualities of stem cell biologics play an essential role in ameliorating the neuroinflammation that remains during the progressive phase of stroke [134]. Stem cells protect the brain against progressive stroke-induced inflammation by stimulating anti-inflammatory cytokines and attenuating the expression of pro-inflammatory cytokines [134]. This area includes pro-inflammatory mediators such as IL-1B, tumor necrosis factor-α (TNF-α), and IL-6 [31]. These cytokines result from activated damage-associated molecular patterns (DAMPs) from cells of the innate immune system such as phagocytic microglia and neutrophils that migrate through the newly permeable BBB in response to stroke [31,33]. This innate immunological response recruits the adaptive immune system cells that cause more damage and cell death within the penumbra [72]. Combating this harsh environment paves the way for rehabilitation modalities to provide a more curative effect. Interestingly, this natural process of neural inflammation aids in the recruitment of NSCs in the acute phase through chemotactic signaling but becomes chronically aberrant [36,135,136]. MSCs aid in the rescue of the neurons in the ischemic penumbra by attenuating monocyte chemoattractant protein-1 (MCP-1) [31,72]. On the one hand, this suppresses TNF-α and IL-6 via VEGF and on the other hand increases secretion of anti-inflammatory cytokines such as IL-10 and transforming growth factor-β (TGF-β) [137].

Likewise, exogenous stem cells also protect the ischemic brain by suppressing apoptosis and enhancing endogenous neurogenesis, therefore stimulating self-repair [138]. In summary, the mechanisms underlying stem cell-induced neuroprotection and rehabilitation are multi-faceted, including regenerative, anti-inflammatory, and anti-apoptotic processes. 

### 1.3. Endogenous Repair Mechanisms against Ischemic Stroke

#### 1.3.1. Neurogenic Niches House Endogenous Cells 

As demonstrated in previous discussions, exogenous stem cells also exhibit the capacity to stimulate endogenous repair mechanisms, specifically targeting the neurogenic niches. In the adult mammalian brain, neurogenic niches are located in the SVZ of the lateral ventricle and the SGZ of the dentate gyrus [59,131,132,133]. The neurogenic niches consist of NPCs and vascular tissue, which coordinate to initiate adult neurogenesis [59,133]. Neurogenetic niches also contain neurotrophic elements, proteomes, immune cells (e.g., microglia), and anti-inflammatory cytokines, all of which exhibit regenerative properties [30,134]. Therefore, harnessing the rehabilitative capacity of the neurogenic niches for the treatment of stroke may bolster post-stroke neurogenesis and patient recovery. 

#### 1.3.2. The Role of Neurogenesis and Angiogenesis in Stroke Recovery 

The rehabilitation of neurovascular networks following ischemia is critical for long-term patient recovery [59]. Neurogenesis and angiogenesis bolster brain plasticity post-stroke, and thus promote rehabilitation [59,135,136]. After a stroke, the brain experiences damage in the ischemic core and the penumbra. Cells within the core immediately die due to necrosis, yet cells in the penumbra may survive for a limited time. These cells can be rescued with enough blood flow restoration and accurate timing [137]. Notably, the brain has an innate capacity to renew injured neurons and curb further cell death in the penumbra via endogenous repair mechanisms, such as neurogenesis and angiogenesis. An ischemic event triggers neurogenesis and angiogenesis in the SVZ and homing of neuroblasts from the SVZ to the ischemic boundary zone [59,131,132,133,136,138,139]. At the site of ischemic injury, these neuroblasts then develop into mature neurons to replace damaged cells [59,132,136,138,139].

Additionally, angiogenesis after ischemic stroke promotes the release of several growth factors, which aid in neuroprotection of living tissue in the penumbra and migration of neuroblast cells to the ischemic insult [59,140]. Secretion of inflammatory cytokines/chemokines and neurotrophic elements also help proliferate NPCs [136,141,142,143]. NPCs then travel to the ischemic border zone and alleviate neuronal loss [144]. Altogether, the brain demonstrates an intrinsic capacity to promote neural regeneration post-ischemia by stimulating adult neurogenesis [59]. Enhancing this recovery process serves as a promising therapeutic strategy against ischemic stroke [59].

#### 1.3.3. White Matter Repair Mechanisms

The brain exhibits innate repair mechanisms that target white matter damage. White matter injury induced by ischemic stroke generates significant injury to oligodendrocytes, axons, and myelin sheaths [65,145]. Stroke-induced white matter damage entails rapid myelin deterioration [145,146,147] and axonal loss in the ischemic infarct [148]. However, white matter injury outside the ischemic core can be rehabilitated [145]. Remyelination, dendritic sprouting, and axonal outgrowth aid in the recovery of stroke-induced white matter injury and impaired neuronal circuitry [59,135]. White matter repair mechanisms also include the regeneration of damaged oligodendrocytes [149]. After ischemic stroke, NSCs in the SVZ differentiate into OPCs, which travel to white matter tracts located in the corpus callosum, striatum, and the ischemic region [59,150]. In these areas, the OPCs develop into mature oligodendrocytes that secrete myelin [59,150]. Therefore, therapeutic interventions that heighten endogenous repair processes, such as oligodendrogenesis and remyelination, stand as a robust treatment strategy to target stroke-induced white matter deficits [65,149].

#### 1.3.4. Bystander Effect

After the transplantation of stem cells, stem cells can operate by (i) causing cell replacement or (ii) secreting paracrine factors, known as the bystander effect [55,151]. Transplanted MSCs and NSCs produce several growth factors, as well as neurotrophic factors, such as BDNF, VEGF, and EGFs [11,152], which may play a role in enhancing endogenous neurogenesis [11,153]. Interestingly, BDNF and nerve growth factor (NGF) promote NSC proliferation and stimulate NSC differentiation into oligodendrocytes in vitro [153]. In addition, treatment with both BDNF and EGF promotes host cell proliferation in both the SVZ and ipsilateral striatum following hypoxic injury, offering a potential combination therapy to treat stroke [11,154]. Alone, IV delivery of BDNF can promote neurogenesis and NPC movement from the SVZ to the injured site, culminating in alleviated sensorimotor impairment [11,86]. Additionally, VEGF stimulates angiogenesis, which is critical for the functionality of the neurogenic microenvironment [11]. In short, the release of these secretomes improve the neurovascular microenvironment by recruiting endogenous cells towards the injured site and promoting neurogenesis. 

### 1.4. Therapeutic Promise of Stem Cells

In conclusion, stem cells have risen as a potential therapy for stroke, exhibiting robust efficacy for regeneration and neuroprotection. Stem cell treatment can foster rehabilitation via exogenous mechanisms such as cell replacement, amelioration of neuroinflammation, and attenuation of apoptosis. Interestingly, stem cells can also promote neurovascular restoration post-stroke via endogenous stem cell recruitment. Exogenous stem cells can stimulate neurogenesis in the neurogenic niches and also direct the migration of endogenous progenitor cells to the ischemic region via chemotaxis and biobridge formation.

## 2. Remodeling of the Stroke Tissue Microenvironment within the Brain 

Neurodegenerative diseases, stroke, primary and metastatic brain tumors, and TBI are neurological disorders affecting humans across the globe. Disease progression is characterized by alterations in the microenvironment of the brain [155]. More specifically, stroke entails primary and secondary cell death pathways, altogether creating a harsh microenvironment. This limits the survival and growth of exogenous and endogenous stem cells, subsequently limiting brain repair [155]. In order to reduce this limiting factor, novel strategies can be designed to enhance the “regenerative” microenvironment and may promote an improvement in brain repair. 

### 2.1. Exogenous Stem Cell Remodeling

Modulation of stem cell adhesion and chemokine receptors may increase therapeutic efficacy in a clinical setting. Human BM-MSCs subject to cationic molecule polyethyleneimine (PEI) treatment retained their capacity to differentiate, immunomodulate, and survive. Augmented CC chemokine receptor 4 (CCR4) expression decreases adhesion capabilities in vitro by blocking adhesion receptors. Furthermore, IV administration of PEI-MSCs in a rodent model increases homing rates to the brain and decreases the presence of PEI-MSCs in the lung vasculature. A cell’s ability to adhere and migrate within the local microenvironment influences therapeutic capabilities, as shown in a mouse glioblastoma model with non-PEI-MSCs conversely displays a heightened tumor migration [156]. Cell-free exosome therapy through the paracrine effect prevents difficulties when compared to cell therapies. Although this is a promising therapy for the future, there are still some issues between the in vivo and in vitro microenvironment and culture conditions that affect the paracrine effect of stem cells [157].

MSC and local cooling infusion demonstrates the ability to reduce infarct volume, while combination therapy provides even more functional benefits. An increase in Miro1 expression results in higher rates of mitochondrial transfer and greater neuronal cell viability and ATP production. Therapeutic hypothermia presents a viable therapeutic option for treating ischemic stroke. Therapeutic hypothermia can enhance MSC mitochondrial transfer-mediated neuroprotection while causing an increase in the production of Miro1 and a decrease in reactive oxygen species (ROS) [158]. MSCs have also shown significant efficacy as a therapy for cerebral ischemia/reperfusion (I/R) injury. The harsh microenvironment associated with cerebral I/R shows MSCs may be associated with poor survival rates of engrafted cells. CUE domain-containing 2 (CUECD2) mediates antioxidant capacity in cardiomyocytes eliciting protective effects against ischemia. When introduced to the ischemic brain, CUECD2 alone substantially decreases neuronal apoptosis and oxidative stress in vitro. Furthermore, siRNA-CUECD2-enhanced MSCs promote efficacy by improving I/R-associated neuronal injury and tissue damage compared to non-CUECD2-modified MSC populations. Specifically, CUECD2-modification magnifies MSC’s anti-inflammatory and antioxidant capabilities in co-cultured neurons via downregulating NF-kB and increasing glutathione peroxidase-1 concentrations. These observations provide an avenue to maximize the clinical efficacy of MSCs [159]. 

A novel approach to alter the surface of poly lactic-co-glycolic acid nanoparticles (NPs) by coating NSC membranes reveals an increase in brain penetration. Furthermore, coating NPs with the membrane of NSCs overexpressing CXCR4 enhances these positive effects due to the chemotactic interaction of SDF-1 and CXCR4, which are augmented in the ischemic microenvironment. Moreover, CXCR4-overexpressing membrane-coated NPs strengthen the anti-inflammatory effects of the stroke treatment glyburide. This novel strategy enhances drug efficacy in the stroke brain and warrants further investigation as a potential therapy in a clinical setting [160]. 

NSCs preconditioned with adjudin facilitate NSC survival in the hostile microenvironments of the stroke infarct area. This treatment evokes a decrease in infarct volume, as well as neurobehavioral deficiency. Pretreatment with adjudin also promotes survivability in a hydrogen peroxide-induced cell death model in vitro through the inhibition of oxidative stress and activation of Akt signaling [161].

NSC transplantation may provide therapy for ischemic brain injury, but the hostile microenvironment in the ischemic brain sets barriers that need to be overcome before using such treatments. bFGF plays a significant neuroprotective role, and bFGF gene-modified NSCs may improve neurological function. Modified infused NSCs exhibits survival and proliferation of NSCs 24 h after cerebral artery occlusion. Furthermore, IV infusion of NSCs improves functional recovery, and bFGF promotes stem cell differentiation into mature neurons [162].

Interferon-γ (IFN-γ), a pro-inflammatory cytokine, can protect stem cells during the inflammatory response and stimulate the neuronal differentiation of these cells. Combined IFN-γ treatment with stem cells increases neurogenesis in vivo and promotes neurological repair [163]. The inflammatory response generated during ischemic stroke affects functional and structural recovery. IFN-γ causes inflammatory effects on native stem cells but does not affect the proliferation of the NSCs. This illustrates the NSCs role in recovery after ischemic stroke in rats by attenuating the inflammatory response [164]. 

NSC cells pretreated with sodium butyrate and nicorandil reduce infarct size and promote cell survival in a rat stroke model. Preconditioned cells with small molecules promote BDNF levels, and higher apurinic/apyrimidinic endonuclease 1 and phosphoinositide 3-kinase levels, correlated with neurite outgrowth. Furthermore, donor cell survival increases and stroke injury site declines in glial fibrillary acidic protein (GFAP) +cells, proinflammatory cytokines, and CD68. An increase in anti-inflammatory mediators and suppression of pro-inflammatory mediators in pretreated NSC cells contributes to inhibiting the inflammatory response. Neurological function remains stable in the preconditioning treatment, illustrating that a small molecule preconditioning approach improves survival of stem cells and inhibits microglial activation [165].

### 2.2. Endogenous Stem Cell Remodeling

Extracellular vesicles (EVs) from stem cells interact with immune recipient cells to provide therapeutic effects in pro and anti-inflammatory environments. EVs tend to act with recipient microglia, such as in tumor microenvironments where EVs regulate immune dampening through interaction with microglia. The function of EVs can be controlled and applied therapeutically in proinflammatory neurological disorders including stroke, Alzheimer’s disease, and PD. EVs extracted from stem cells demonstrated the ability to attenuate proinflammatory responses [166]. Umbilical cord MSCs (UC-MSCs) are regarded as a potential therapy for ischemic stroke. However, poor survival rates are observed in vivo due to the harsh inflammatory microenvironment of the ischemic brain. EVs derived from ischemia-injured N2A neurons co-cultured with UC-MSCs indicate a significant increase in oxidative stress and apoptosis following OGD/R. Conversely, knock-down of Rab27a shields UC-MSCs from OGD/R injury. Furthermore, hypoxic preconditioning of UC-MSCs enhances paracrine mechanisms and augments cell survival rates. Thus, hypoxic preconditioning improves UC-MSC survival while EVs from insulted N2A cells exacerbate the adverse OGD/R effects on UC-MSCs [167]. 

NSCs have demonstrated promising therapeutic potential in promoting neurological repair. NSCs provide beneficial effects on the stroke brain due to their cell replacement, paracrine, inflammatory regulation, and neuroprotection capabilities [48]. An all-human endothelial cell and NSC co-culture model used to analyze gene expression in order to determine how NSCs interact with the vasculature after transplantation shows that co-culturing has a significant effect on the gene expression of both NSCs and ESCs when cultured alone. This reveals that monoculture models alone provide an inaccurate expression profile when investigating cell signaling effects within the brain. In contrast, in vitro co-culture models allow for a realistic mechanistic understanding of NSCs in the brain, taking into account the interaction with surrounding tissue [168]. Oxygen concentrations influence the brain microenvironment and act to promote NSC neurogenesis. MCAO rats put through intermittent hypoxic preconditioning (HPC) exhibit a significant decrease in axonal and neuronal injuries, a reduction of apoptosis, and enhanced differentiation and migration [169]. Furthermore, the therapeutic effects of NSCs are limited in the microenvironment of ischemic regions rich in ROS. Following TBI, an NT3-chitosan injection in the lesion site effectively promotes NSCs to proliferate and migrate towards the lesion site through three main actions: pro-neurogenesis, anti-inflammation, and pro-revascularization. By creating a non-hostile microenvironment, the NSCs are capable of executing neural repair [170]. NSCs showing overexpression of the SUMO E2-conjugase Ubc9 demonstrate resistance to OGD/R. The NSCs also show enhanced neural differentiation and increased survival among mice with MCAO [171]. Moreover, NSCs can amplify their therapeutic effects through gene transfection augmenting BDNF expression, resulting in a higher rate of survival and a more rapid and efficient functional reconstruction [172].

Human-IPSC-derived neural progenitor cells (hNPCs) are a promising therapy to treat neurological diseases. However, there has been little investigation of the relationship between the electrical and physical microenvironment and hNPC function. Electrical stimulation of hydrogel-immobilized hNPCs alters hNPCs genes involved in metabolic pathways and cell proliferation upregulates neurotrophic factors correlated with nerve regeneration, synaptic remodeling, and cell survival. These findings indicate that electrical stimulation modifies hNPC properties and may be beneficial to provide a novel therapy for neurological disease [173]. 

When the BBB is disrupted, it alters the composition of the brain’s microenvironment by allowing plasma proteins in. Fibrinogen activates the BMP signaling pathway in the OPCs, as well as inhibits remyelination. Fibrinogen also supports the phosphorylation of Smad1/5/8 and inhibits the OPC differentiation into myelinating oligodendrocytes, which supports astrocytic fate in vitro. Fibrinogen and the BMP increase demyelinating multiple sclerosis lesions, while the depletion of fibrinogen decreases BMP signaling, and promotes remyelination in vivo [174].

Various emerging stroke therapies target molecules and cells in different phases of an ischemic stroke. By utilizing immunohistochemistry, pathophysiological changes in the brain microenvironment are revealed at multiple phases following a stroke. The presence of NeuN+ neurons, GFAP+ astrocytes, Iba1+ microglia, and cell death-related molecules signal the progression of damage to the brain. 

### 2.3. Use of Biomaterials, Drugs, Photo- and Optogenetics 

The mechanisms in which drugs and biological compounds bolster cell therapy include facilitating BBB stem cell penetration, dampening inflammation, promoting neurogenesis, and enhancing angiogenesis [12,13]. In MCAO murine models, the use of statins exhibit an influential decrease of infarcted volume and lower expression of NLRP-1 and NLRP-3 genes [175,176]. Erythropoietin and metformin reduce hypoxia and induce angiogenesis by inhibiting hypoxia-inducible factor-1α (HIF-1α) expression and preventing nitrate stress, respectively [177,178]. Following that pattern, granulocyte-colony stimulating factor administration may have a synergic effect with stem cell therapy in stroke by reducing proinflammatory expression and thereby dampening the post-stroke inflammatory environment that inhibits neuroregeneration [179]. Another common drug under evaluation in umbilical cord blood stem cell transplantation is mannitol [12,180]. Mannitol’s hyperosmolar property increases BBB permeability, which may enhance stem cell penetration by reducing physiological impediments [181,182]. Likewise, concomitant administration of mannitol and temozolomide may enhance the BBB permeability by inhibiting endothelial tight junctions [183]. 

Mouse-IPSC-derived NPCs were investigated to uncover whether selective excitation may restore an activity-enhanced microenvironment to promote regenerative benefits after stroke. Transduction of iPSC-MSCs with luminopsin 3 and activation with light or coelenterazine (CTZ) displays increased levels of synapsin-1, postsynaptic density 95, BDNF, and SDF-1, all promoting neuronal growth in vitro. In vivo, transplanted LMO3-iPSC-NPCs and CTZ stimulation leads to axonal myelination, synaptic transmission, and thalamocortical connectivity, resulting in functional recovery. Furthermore, improved neural network connections are seen in the peri-infarct region in mice treated with LMO3-iPSC-NPC/CTZ. Results reveal that exciting transplanted cells provides a novel therapeutic avenue for stroke [184].

New progress in biomaterials and bioprinting suggests that regenerative medicine for neurological applications may be a viable option for treating neurological disorders. Loss of tissue volume during a TBI could be replaced with artificial tissue created with bioprinting technology [185]. Hydrogel biomaterials show the ability to mark biomolecules, adhesion motifs, growth factors, and other cues for stem cell encapsulation. Application of hydrogels in stroke as a favorable microenvironment for stem cell transplantation is a potential target for stroke treatment. Efficient hydrogel systems are constructed and used in neurological applications [186]. Regenerative medicine is used to provide a favorable microenvironment for stem cells to develop. Physiological and pathological cavities, such as lateral ventricles and stroke lesions, may act as a viable gateway for developing stem cells. Hyaluron, a naturally occurring polymer, can function along with enzymes as an approach to dissolve the connective tissue that acts as a barrier between the scaffold and the host, thus resolving the limited migration of transplanted cells [187]. Additionally, hyaluronic acid and methylcellulose hydrogels demonstrated improved survival and integration of stem cells [188,189]. Furthermore, hyaluronic acid has been related to angiogenesis induction and cell proliferation [190]. In contrast, methylcellulose promotes axon connections and neuron regeneration [188,190].

By studying the biomaterials composition influence on stem cell transplantation therapy, it has been observed that material stiffness confers stem cells’ different properties. Stiffer substrates direct NSCs to astrocyte differentiation, while softer substrates lead NSCs to neurogenic differentiation [191,192]. Chemical composition proves essential for potential beneficial outcomes, particularly in injectable hydrogels development [192]. 

Myelination is crucial for efficient neuronal signaling, making remyelination a key therapeutic strategy for diseases such as multiple sclerosis, stroke, and spinal cord injury. Biomaterial technology has the ability to aid OPCs and oligodendrocyte development and regenerate healthy myelin sheaths in damaged CNS tissue. Biomaterial scaffolds created from ECM can increase transplanted cell survival by altering the fate of OPCs and oligodendrocytes [193]. 

MSC-derived EVs show remarkable therapeutic promise in CNS repair. EVs lack the risks involved with stem cell graft-host rejection and promote recovery by enhancing intercellular communication on the nanoscale level via the secretion of factors associated with injury improvements. Moreover, utilizing biocompatible injectable hydrogels coupled with EVs may bolster their therapeutic capabilities by providing the stroma necessary for brain rehabilitation [194]. 

Ischemic brain injury results in altered neural cell metabolism, which causes cell membrane hyperpolarization and intracellular acidosis. Extracellular calcium may hamper pH regulation and sodium bicarbonate transporter activity in the brain injury microenvironment. Removal of extracellular calcium allowed for recovery of pH and rapid depolarization. The recovery phase present in the absence of calcium depends on an electrogenic sodium bicarbonate transporter NBCe1(SLC4A4). These findings indicate that excess extracellular calcium may prevent proper pH regulation in a brain injury microenvironment, suggesting that calcium-sensitive transporters are essential for neuronal proliferation, survival, and NSC differentiation [195].

Neurogenesis and angiogenesis provide a viable target for NVU modeling due to the close network between endothelial cells and NSCs. Further research should work to develop predictable technologies that control the fate of stem cells, provide accurate screening of drugs for the nervous system, and further improve the application of NVU models in a clinical setting [196].

### 2.4. Novel Drug Therapies and Mechanism of Action

Stand-alone drug therapeutics for the long-term management of stroke present promising data. Five pharmaceutical approaches have been identified that share common neuroprotective and regenerative effects post-stroke. These include ligands for 18-kDa translocator protein (TSPO), raloxifene (a selective estrogen receptor modulator), fucoidan (a brown seaweed product), SMM-189 (a CB-2 receptor ligand), and pifithrin (a P53 modulator) [197]. Of these drugs, TSPO, raloxifene, and pifithrin display the most potential for use with stem cells. The discovery of TSPO offers a new approach to diagnosing neuroinflammation, managing apoptosis, mitigating ROS, and regulating gene expression post-stroke [198]. TSPO is a transmembrane receptor located on the outer mitochondrial membrane and facilitates the transport of cholesterol for neurosteroid synthesis in microglia, astrocytes, neurons, oligodendrocytes, endothelial cells, and more. Changes in TSPO expression in different cell types highlight the growing need for an exploration of its role in neuroinflammation. TSPO ligands show promising neuromodulatory effects by increasing steroid synthesis, leading to neuroprotective and neuroregenerative effects [199]. Ligands such as PK11195, Etifoxine, Emapunil, and 2-Cl-MGV-1 have been shown to decrease levels of pro-inflammatory cytokines such as IL-1B, IL-6, TNF-α, and IFN-γ. However, whether these ligands induce neuroprotection because of direct effects on neurons, modulation of the microenvironment by glial cells, or recruitment of endogenous stem cells remains in question. Decreases in TSPO in astrocytes correlate with decreases in the A1 pro-inflammatory phenotype in the brain post-stroke. Additionally, decreased levels of TSPO promoted the M2 anti-inflammatory phenotype of microglia, leading to more significant expressions of IL-4, IL -13, BDNF, and IGF [198]. Plasma TSPO levels are also known to be intimately linked with disease progression and worse functional outcomes post-stroke [200]. The development of TSPO ligands and radiolabeled markers for TSPO expression provide two avenues of function for use in post-stroke patients. Radiolabeled TSPO will allow for an accurate distinction between inflamed tissue for adequate diagnostics of neurodegeneration and timing for stem cell transplant. TSPO ligands have been shown to increase levels of anti-inflammatory molecules in tandem with decreased levels of pro-inflammatory molecules, hinting at a possible additive or synergistic application when used with stem cells.

Another promising drug is raloxifene due to its protective effects. In animal models, raloxifene displays curative effects post-stroke. The mechanism by which raloxifene exerts its effects is closely related to the other five drugs and can be categorized into genomic and nongenomic ways. From a genomic perspective, the drug modulates protein expression through nuclear estrogen receptors, namely ERα and ERβ. Raloxifene also modulates ROS, mitochondrial function, glucose metabolism, apoptosis, and cholesterol levels in a favorable way to promote healing and neuronal survival [197]. Interestingly, its ability to elicit robust neurogenesis in the SVZ of rats compared to placebos offers an exciting potential application with stem cells to increase endogenous neurogenesis [201].

Pifithrin-alpha is a p53 inhibitor. This drug targets the p53 tumor suppressor protein responsible for cell cycle arrest and ultimately apoptosis. p53 is activated under conditions that induce DNA damage such as ROS. Inhibiting this protein resulted in anti-apoptotic activity in neurons post-stroke and should be investigated in tandem with stem cells to aid in the rescue of neurons exposed to the harsh post-stroke microenvironment and subsequent reperfusion [202].

### 2.5. The Future of Therapeutic Approaches 

One therapeutic approach to rehabilitate the stroke brain may be finding the most effective transplantable type of exogenous stem cells against ischemia. Alternatively, modifying the exogenous stem cells via hypoxic cell culture exposure or genetic modification to make them more resistant following their transplantation to the stroke microenvironment may serve as equally feasible strategies. Furthermore, enhancing the survival of endogenous stem cells in neurogenic niches and facilitating their migration to the ischemic tissues acts as another modality in combating the harsh stroke microenvironment. Drug therapies or feverish engineering, such as RNAs and ontogenetics, can improve survival and direct the migration of endogenous stem cells. Finally, biomaterials can nurture both exogenous and endogenous stem cells towards the repair of the stroke brain. Altogether, these treatments, either as stand-alone or in combination, display appealing therapeutic features in harnessing enhanced regenerative medicine for stroke. 

## 3. Remodeling of Stroke Tissue Microenvironment outside the Brain 

Combination treatments of stem cell transplantation and enriched environment/rehabilitation therapy may prove beneficial for stroke. Indeed, rehabilitation therapy stands as the default treatment for stroke patients. The addition of stem cell therapy to rehabilitation may accomplish the desired goal of creating a conducive host tissue microenvironment while teaching the graft-host new circuitry to aid the brain repair process. This section will discuss: (1) Prominent modalities of rehabilitation in stroke and how they aid in functional recovery; (2) How stem cells with rehabilitation may provide a synergistic or additive mechanism for functional recovery.

### 3.1. How Do Prominent Modalities of Stroke Rehabilitation Aid in Functional Recovery? 

Motor dysfunction results from a range of neurological diseases and creates enormous social and economic burdens for patients. Patients who have motor dysfunction have trouble performing daily activities and suffer from limited mobility. These morbidities cause a third of stroke patients to suffer from permanent motor deficits that affect their activities. For this reason, patients should seek some sort of motor rehabilitation therapy. Although there are functional improvements with motor rehabilitation, residual disability and neurological deficits remain a concern for physicians [203]. 

Understanding brain plasticity and the development of artificial intelligence play a role in functional motor recovery. Brain plasticity is the ability of the brain to adapt to change and environmental stimuli. This plasticity is stimulated by therapeutic treatment, brain damage, and experiences leading to the reorganization of its function, structure, and connections [203,204].

Current data show that structural and functional brain plasticity mechanisms act on different aspects of the development and function of the brain, depending on the enriched environment (EE). Therefore, in animals, this concept implies that social stimuli and new objects influence the changes in the brain structure and function during learning. Cognitive processes correlate with molecular mechanisms of synaptic transmission, and they represent a possible target for the action of environmental factors in the brain under physiological conditions. EE improves gross neuroglia, sensorimotor function, spatial learning, and memory but is limited to the activity of the animal [205]. Additionally, the theory of EE describes that the neuron-glia relationship is influenced by brain damage [13,206,207,208]. Rats subject to intracerebral hemorrhage show functional improvement and an increase in the survival of neurons [209]. A three-phase paradigm for EE where the EE for rats was changed based on the three phases of stroke revealed changes to the ischemic microenvironment including significantly improved survival of cortical and striatal neurons, improved cerebral blood flow, decreased BBB damage, and increased angiogenesis via modified VEGF, Angiopoietin-1 and Angiopoietin-2 compared to standard housing rats [210]. EE provides an animal model for rehabilitation measurable through standardized behavioral assessments not common in traditional human rehabilitation due to the lack of standardization and subjectivity of clinician experience [203].

A new intelligent rehabilitation platform focused on conventional rehabilitation treatments has been developed to assess the recovery process through training. Several advanced smart technologies such as virtual reality (VR), brain-computer interfaces (BCI), magnetic stimulation of neural circuits, and robot-assisted therapy are currently being developed to improve recovery in patients with motor dysfunction [211].

A precise and quantitative evaluation system is necessary to provide an efficient allocation of treatments for motor dysfunction to predict the functional status of patients. Electromyography and electrophysiology, motor evoked potentials, and typing techniques can be used clinically to assess muscle condition [211]. Neurological biomarkers derived from neuroimaging technologies have more prognostic and predictive value for motor recovery than clinical behavioral biomarkers [212].

#### 3.1.1. How Does Motor Rehabilitation Facilitate Functional Recovery?

Motor recovery is a complex process related to functional restoration in neural tissue and the ability to perform movements [203]. For this reason, the focus on rehabilitation of neuromuscular functions is rooted in the establishment of sensation-movement neuronal circuits [213].

Currently, restriction-induced therapy [214], motor imaging, bilateral training, mirror therapy, and treadmill training plus body weight support [203,215] are standard rehabilitation therapies that help improve motor ability. Thus, a combination of repetitive task-specific training therapies is the gold standard for motor rehabilitation after stroke [203,216]. Task-specific training induces adaptive neural plasticity, leading to functional motor recovery and synapse formation leading to cortical reorganization in relation to brain regions that are targeted [216,217]. Still, one-third of patients suffer from permanent deficits, and it is essential to seek new rehabilitation alternatives.

#### 3.1.2. How Does Functional Electrical Stimulation (FES) Aid in Functional Recovery?

Somatosensory information is necessary to improve motor performance and effective motor learning. FES shows a potential to increase the effect of sensory inputs afferent to the central nervous system, facilitating the induction of greater excitability of the motor cortex [218,219]. FES works to recruit Golgi tendon organs and activate muscle spindle feedback circuits, thus eliciting motor control [220]. FES-induced plastic changes last between 20 to 110 min and can be integrated into a conventional stroke rehabilitation program to improve motor recovery in stroke patients. The therapeutic effects of rehabilitation are promising, and it is a good treatment option for motor rehabilitation [221]. The outcome of FES therapy in stroke patients is dependent on time after stroke, functionality, corticospinal tract damage, and location of stroke [222]. Chronic stroke patients exhibit cortical excitability at the contralesional site following FES therapy [222]. Stroke patients with less overall impairments exhibit cortical reorganization at the ipsilesional sites leading to better improvements following FES therapy [222]. 

#### 3.1.3. How Does Functional Magnetic Stimulation Assist in Stroke Recovery?

Neuromodulation technology acts on transmitting CNS signals and inhibiting, exciting, and regulating neural network activities, which generates therapeutic effects [223]. Transcranial magnetic stimulation (TMS) is widely used since it is safe and acts on brain metabolism and nerve conduction, thus modifying the state of tissue excitability [224].

Motor functional performance correlates with the imbalance of interhemispheric inhibition between the contralesional and ipsilateral hemispheres after stroke [225]. Reducing excessive inhibition from the contralesional to the ipsilesional hemisphere enhances functional motor recovery. Low-frequency TMS (<1 Hz) is applied to the unaffected side and used to suppress local neural activities. Repeated high-frequency TMS (>10 Hz) is applied to the affected side to activate neural activities, increase brain plasticity, and improve functional reorganization in the affected hemisphere after stroke [203,225,226].

#### 3.1.4. How Does Biofeedback-Based Rehabilitation Training Aid Functional Recovery? 

Interventions based on biofeedback are useful in motor rehabilitation. This modality provides information from the subconscious body processes to consciously improve the movement process [227]. It consists of continuously monitoring a neurophysiological response and these measurements are presented as auditory or visual representations to the individual. The individual perceives the information and can modify their performance to achieve better results based on quantifying the muscular state and the intention of activity to promote a comprehensive improvement of movements. It is used in stroke rehabilitation because it improves retention of learned skills and improves balance in patients [228,229,230].

Neurofeedback therapy (NTF) is a specialized form of biofeedback characterized by circumscribed neural activation and provides a visual representation of patient measurements for patients to achieve real-time self-modulation in the motor process in stroke rehabilitation [231,232,233]. NTF influences neuroplasticity by combatting wave activity that has been slowed due to damage following a stroke by presenting visual and auditory stimuli to the patient and training the patient to adapt their brainwave function to meet threshold targets via feedback signals [234]. EEG changes at resting state following therapy indicate improvements in behavior indices as well [234].

#### 3.1.5. How Does Robot-Assisted Therapy Facilitate Functional Recovery? 

Robot-assisted therapy has been developed over the past decade to facilitate independent motor rehabilitation. Devices used for motor training include end-effector and exoskeletal types [235,236]. Exoskeleton-type devices have robot axes aligned with the user’s anatomical axes, which help control individual joints better and improve movements. In contrast, end-effector robots have the advantage of being easy to use but suffer from limited management of the proximal limb joints [236]. 

A real-time, two-axis mirror robot system was developed for conventional mirror therapy with a closed feedback mechanism to control the real-time movement of the hemiplegic arm. The advantage of this therapy is the ability to offer high-intensity, high-dose training, making it easy for patients with motor disorders to use and making it a more effective treatment than conventional therapy. This therapy is currently used in stroke rehabilitation in combination with traditional therapy to increase activity in the upper limb and activate cortical activity at rest and during therapy [236,237,238,239,240]. These two therapies used together induce a larger recruitment level in central areas and the periphery, while showing a higher activity of motor neurons via a direct excitatory feedback loop to the cortex from the basal ganglia or an indirect excitatory pathway influenced by low dopamine pathways [241]. 

#### 3.1.6. How Does BCI Facilitate Functional Recovery?

BCI is a new technique for the rehabilitation of motor control and neural function with the aim of motor rehabilitation [242,243]. BCI is a system with the ability to measure brain activity and convert it into artificial output that restores, replaces, and improves central nervous system output; therefore, it drives external devices [244]. BCI encodes motor information in the central nervous system and builds a brain-machine feedback loop that assists patients in regaining limb control and motor reconstruction, thus serving as rehabilitation therapy in cerebrovascular accident patients. Long-term use of BCI aids stroke patients in recovery of motor function in the upper extremities by reactivation of their musculature and improvement of the operation of the ipsilesional hemisphere [242,243,245]. BCI may also improve structural reorganization in the brain through repeated motor planning and execution and increase ipsilesional cortico-subcortical connectivity with one another [242]. Repeated tasks aimed at increasing connectivity in damaged areas may also improve and strengthen normal motor or cognitive circuits by re-establishing cortical activity and proprioceptive feedback [246]. 

#### 3.1.7. How Does VR-Based Rehabilitation Improve Functional Recovery?

VR systems have been applied in neurological diseases as they provide a multidimensional experience within an immersed, non-immersed, and semi-immersed perspective. This helps users interact with simulated virtual environments in stroke rehabilitation environments and have a measurable recovery [247,248]. Stroke VR systems capture the movements of patients, which the computer displays in a process called motion visualization. Repeated and intentional VR has the ability to improve cortico-cortical motor and premotor connectivity via a feedback system to learn and reduce errors via visual feedback [249]. This motion visualization contributes to the observation of limb movement and activates the mirror neuron system in the area of the frontoparietal cortex [250,251,252].

#### 3.1.8. How Does Non-Motor Rehabilitation Assist in Functional Recovery?

According to a systematic evaluation of database target studies evaluating individuals, fixed models show a significant improvement in motor outcomes. Non-motor aspect domains show the importance of holistic assessments targeting and stimulating personal patients’ profiles. Additionally, the non-motor symptom scale of the quality of life, activities of daily living, motor complications, and other parameters significantly improve proportionately [253,254,255].

Sensory information plays a vital role in motor rehabilitation after a stroke since no motor therapy is considered purely motor. Meaningful motor function involves sensory integration of information to varying degrees. Somatosensory information supplies important sensory details for the motor system. The literature reports that neurophysiological mapping studies have shown that afferent muscle stimulation drives neurons in the primary motor cortex and involves the somatosensory process through functional and anatomical connections with the primary and secondary somatosensory cortex, as well as with the sensory thalamus [256,257].

After a lesion to the motor cortex, the brain activates compensatory methods to maintain motor control by reassigning sensorimotor interactions through the recruitment of primary sensory cortices, secondary motor areas, and areas of higher-order association involved in sensorimotor transformations. Therefore, before stroke, it is crucial to restore sensory processing and sensorimotor interactions to regain function. Sensory therapies facilitate learning and compensation for lost motor function. However, most strategies focus on motor functions because it is the gold standard of rehabilitation therapy for stroke [257,258,259,260].

### 3.2. How Do Stem Cells with Rehabilitation Offer an Additive or Synergistic Functional Recovery?

Both stem cell therapy and rehabilitation have a wide therapeutic window making them excellent candidates for long-term treatment post-stroke [205]. The adult mammalian brain is more plastic following injury, indicating a window of opportunity to intervene with therapeutics [261]. This combination therapy is relatively unexplored; however, animal studies show promise [70]. 

EE provides a standardized and reproducible animal model for rehabilitation, assisting researchers in understanding the benefits of rehabilitation [203]. Traditional rehabilitation methods such as physical therapy, physiotherapy, occupational therapy, and speech therapy have proven beneficial in functional recovery. Still, they vary from patient to patient, lack standardization, lack in-depth research on cellular mechanisms, prove hard to measure, are dependent on the experience of clinicians, and often fall short of full functional recovery for patients [203,262]. After rehabilitation, the functional recovery observed in rats and patients may be due to neuroplasticity and compensation by healthy neurons rather than neural repair and regeneration [35,263]. Stem cells could one day fulfill this gap in regeneration and assist in restoring lost neural circuits and structure with rehabilitation as a reinforcer of these circuits. 

#### 3.2.1. How Does Enriched Housing in Tandem with Stem Cells Enhance Functional Recovery? 

Rats housed in an EE and subject to voluntary exercise enhanced transplanted stem cell migration to the area of infarct and improved survival of the transplanted stem cells. EE increases the number of endogenous neural progenitor stem cells in the SVZ of rats post-stroke, suggesting another exogenous method of stem cell recruitment [264,265]. MCAO rats subjected to either EE, stem cell transplant, or the combination of both within a week of the insult results in an increased functional recovery of the affected limb with combination therapy compared to either method alone [205]. In a follow-up preclinical study, MSCs were again evaluated with EE after MCAO and confirmed the significantly improved behavioral outcome seen with combination therapy. The rats were given MSCs two and seven days after stroke with continuous EE and evaluated on day 46 with substantial functional recovery and the presence of long-term microglia in the penumbra [70]. Replacement of lost neurons will not be enough to aid in functional recovery. Cell therapy may stimulate plasticity mechanisms and facilitate the structural reorganization to allow rehabilitation-induced stabilization of newly formed neural circuits. Physical exercise after MCAO mediates enhanced functional recovery by increasing the NSC movement and distance to the SVZ by upregulating migration and immunological factors [266].

Neuronal precursors and NSC may promote repair after a cortical stroke by secreting trophic factors that aid in cell survival. EE promotes NPC migration to the injury site. However, long-term survival of these precursors is only seen in the ischemic striatum [267].

#### 3.2.2. How Does an EE with Stem Cells Work Together to Modulate the Microenvironment?

EE induces an increased level of proteins related to maintaining plasticity in the brain, in which transplanted SVZ cells are responsive [268]. EE following infarction induced Gadd45b, via BDNF, which led to an increase in the genesis of NPCs in the SVZ [269]. BDNF is one of the neurotrophic factors found in higher concentrations after stem cell therapy alone and is likely responsible for rescuing neurons, increasing dendritic density, and preventing glial scars [53]. This activity suggests an additive or synergistic effect between rehabilitation and stem cell treatment since both modalities increase BDNF. BDNF, when exposed to exercise, helps support neuronal growth and differentiation and promotes plasticity [270]. EE increases functional motor recovery, synaptogenesis, and corpus callosum thickness [271]. The corpus callosum thickness may contribute to the prevention of transcortical projection loss or to the increased plasticity seen between the hemispheres [271]. NGF protein levels are also increased following EE, which may indirectly increase MSC proliferation [272]. Additionally, EE alone also positively affected neurovascular remodeling and revascularization through VEGF. MSCs increased vascular remodeling through VEGF and proves that EE has an additive effect through more than just neurogenesis and synaptic plasticity, but in modulating the microenvironment and maintaining homeostasis as well [210].

EE exposure increased GFAP expression in stroke lesioned rats leading to cognitive improvement [273]. Glial cells express this protein during development in the brain and point to a mechanism by which EE promotes astrogenesis and gliogenesis like transplanted stem cells. The same study found that EE has implications for immune modulation by decreasing IL1-β, an inflammatory cytokine found in higher concentrations after ischemic stroke [273]. Neurogenesis may be induced from an EE, via the NF-κB/IL-17A signaling pathway in the SVZ and striatum [274]. Thus, EE reduces inflammation, thereby increasing transplanted stem cell long-term survival [268].

#### 3.2.3. How Do Exercise and Stem Cells Elicit an Increased Therapeutic Effect When Used Together?

In the ischemic penumbra, exercise inhibits apoptosis and potentially prolongs the MSC survival time via an underlying mechanism involving proto-oncogenes [275]. Physical activity in an EE may also increase cell proliferation and neural progenitors in the SVZ and lead to cortical remodeling, as suggested by the increase in synaptic density [265,267]. In transplanted SVZ cells, migration and long-term survival can be attributed to inflammation [268]. However, EE has been shown to increase progenitor proliferation and long-term survival [276]. EE is also beneficial in migration and increases transplanted SVZ stem cells and clusters close to the injury site. Any transplanted SVZ stem cells that do not differentiate into mature neurons may secrete growth factors, contributing to brain plasticity following an ischemic injury [268]. These stem cells may help restore function around an injury lesion by communicating with surrounding cortical regions [264]. More research is still needed to explore combination therapy before translation into clinical research. The timing, dose, and modality for stem cells and rehabilitation are paramount for success and functional recovery [70]. 

### 3.3. Combination Therapy Potential Benefits

Combination treatments consisting of stem cell transplantation and an EE or rehabilitation therapy may prove beneficial for stroke patients (Table 1); indeed, rehabilitation therapy stands as the default treatment for stroke patients. The addition of stem cell therapy to rehabilitation may accomplish the desired goal of creating a conducive host tissue microenvironment while teaching the graft-host new circuitry to aid in the brain repair process. Stem cells, drugs, and biomaterials can be thought of as the building blocks of brain regeneration, creating the infrastructure needed to facilitate new circuitry to lead to a robust functional recovery. This section discussed how EE and rehabilitation strategies enhance stem cell therapy in stroke patients. 

## 4. Conclusions

Stroke remains a leading cause of death and disability worldwide [280]. Unfortunately, there exist limited therapies for stroke. Acute thrombolytic stroke therapy possesses significant clinical benefits; however, due to the narrow therapeutic window, there remains a substantial need for therapies that can be utilized beyond the acute time frame [31]. Endovascular mechanical thrombectomy grants a larger therapeutic window but still requires administration hours rather than days following a thrombotic event [6]. Stem cell therapies represent an exciting potential therapy for ischemic stroke that could significantly extend the therapeutic window of stroke therapy. Preclinical stroke models demonstrate excellent responses to stem cell therapy with improved infarct size, cognitive and motor function, and survival through cell replacement, neuroprotection, bystander effects, neurotrophic factor secretion, ECM remodeling, the biobridge mechanism, neurogenesis, angiogenesis, astrogenesis, synaptogenesis, and oligodendrogenesis [11,12,13,31]. Generally, the therapeutic effects of stem cells in ischemic stroke can be broadly defined as neuroregenerative, where stem cells replace or facilitate the replacement of dead or damaged neuronal cells, and neuroprotective, by mediating inflammation and limiting the degree of brain damage [16,72].

Despite exciting results in preclinical models, clinical trials have failed to maximize the full therapeutic potential of stem cells, with clinical studies demonstrating at best a modest therapeutic effect [9,13]. Fortunately, clinical investigations have established the safety of MSC therapy in stroke patients [16]. Many elements likely contribute to the lack of translation of preclinical evidence to the clinic such as suboptimal dosage, lack of use of clinical-grade cell lines, timing, and route of administration [9,16,93]. With optimization of protocol and further clinical trials, the full potential of stem cell therapy can be realized in stroke patients.

Furthermore, novel therapies designed to improve the regenerative microenvironment of brain stroke tissue may improve brain repair and efficacy of stem cell therapy. Stroke entails primary and secondary cell death pathways that create a harsh proinflammatory microenvironment and the ability of both exogenous and endogenous stem cells to facilitate neuroregeneration [155]. Modifying exogenous stem cells via hypoxic cell culture exposure or genetic modification allows for improved stem cell efficacy. Additionally, strategies designed to enhance endogenous stem cell survival and their migration to ischemic tissue demonstrate potential for improving stem cell therapies. Utilizing various drug therapies and biomaterials as adjuvants for stem cell therapies can ameliorate the typical harsh ischemic brain microenvironment in stroke patients, allowing a more conducive environment for exogenous and endogenous stem cells.

**Figure 1:**
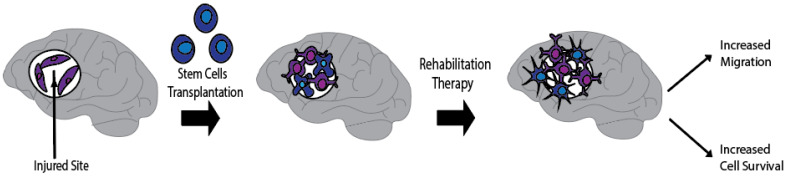
Combination therapy against stroke. Synergistic effects of combined SC transplantation and rehabilitation therapies, such as EE, leads to improved modalities and increased SC engraftment within the stroke injury site. Transplanted SC into the stroke hostile environment may display improved growth, migration, maturation, and neural differentiation when introduced to rehabilitation therapy.

Rehabilitation therapy functions as the default long-term therapy for stroke patients. As the model for rehabilitation in preclinical studies, EE shows the promising benefits of outside strategies to enhance functional recovery. Combining stem cells with EE and rehabilitation therapy may allow for an ideal long-term therapy for stroke patients. Enhancement of brain plasticity is often the target of these therapies. Neuroplasticity is defined as the ability of the nervous system to change its activity in response to intrinsic or extrinsic stimuli by reorganizing its structure, functions, or connections. Neuroplasticity can be enhanced by various therapeutics and depends significantly on the EE. Moreover, the adult brain is more plastic following injury, signifying a window of opportunity for therapeutic intervention [261]. EE promotes improved stem cell survival and migration demonstrating its potential as a combination therapy with stem cells (Figure 1) [267,268]. Motor rehabilitation allows for functional improvements, however, there remain significant concerns for disability and neurological deficits in stroke patients [203]. Various traditional rehabilitation therapies such as physical therapy, physiotherapy, occupational therapy, and speech therapy have proven therapeutic in the functional recovery of stroke patients, however, they lack the potential of full recovery for patients. Multiple novel technological therapies are currently being developed, such as VR, BCI, magnetic stimulation of neural circuits, and robot-assisted therapy, to improve motor function in stroke patients [211]. Combination treatments of stem cells and rehabilitation therapies may prove beneficial for stroke. The addition of stem cell therapy to rehabilitation may accomplish the desired goal of creating a conducive host tissue microenvironment while teaching the graft–host new circuitry to facilitate the repair process.

## Figures and Tables

**Table 1:** Improving the stroke microenvironment via exercise, stem cells, and combination therapies.

Type of Intervention (Exercise/Stem Cells/Exercise + Stem Cells)	Title, Author, Year	Stem Cell Variety	Significant Findings
Exercise	Enforced physical training promotes neurogenesis in the subgranular zone after focal cerebral ischemia [277]	NSCs	Enforced physical training promotes neurogenesis in the SGZ after focal cerebral ischemia [277]
Exercise	Physical exercise regulates neural stem cells proliferation and migration via SDF-1α/CXCR4 pathway in rats after ischemic stroke [266]	NSCs	Exercise improved functional recovery by increasing NSC proliferation, migration from the SVZ and differentiation in the damaged striatum of MCAO occluded rats [266]
Exercise	Postischemic exercise attenuates whereas enriched environment has certain enhancing effects on lesion-induced subventricular zone activation in the adult rat [267]	NSCs	Exercise modulated the stroke induced increase in neural stem cell proliferation in the SVZ early after cortical infarction [267]
Exercise	Different exercises can modulate the differentiation/maturation of neural stem/progenitor cells after photochemically induced focal cerebral infarction [278]	NSCs	Exercise improved neuronal maturation and increased generation of endogenous NSCs [278]
Stem cell transplantation	Stem cell-paved biobridge facilitates neural repair in traumatic brain injury [45]	MSC	MSCs aided endogenous NSCs to the area of infarction, improved behavioral outcomes [45]
Stem cell exosome transplantation	Enhancement of angiogenesis and neurogenesis by intracerebroventricular injection of secretome from human embryonic stem cell-derived mesenchymal stem cells in ischemic stroke model [55]	MSC	MSCs transplantation suppresses inflammation, reduces cell death, promotes angiogenesis, and stimulates neurogenesis [55]
Stem cell transplantation	Activated Mesenchymal Stem Cells Induce Recovery Following Stroke Via Regulation of Inflammation and Oligodendrogenesis [56]	MSCs	MSCs lower overall inflammation, ameliorate potentially toxic environments, and increase neurotrophic factor release, enabling both endogenous NSC survival and function [56]
Exercise and stem cell transplantation	Treadmill exercise enhances therapeutic potency of transplanted bone mesenchymal stem cells in cerebral ischemic rats via anti-apoptotic effects [275]	MSCs	Treadmill exercise enhances the therapeutic potency of MSCs by improving neurological function and possibly inhibiting the apoptosis of neuron cells and transplanted MSCs [275]
Exercise and stem cell transplantation	Synergic Effects of Rehabilitation and Intravenous Infusion of Mesenchymal Stem Cells After Stroke in Rats [262]	MSCs	Both combined therapy and MSC infusion reduced lesion volume, induced synaptogenesis, and elicited functional improvement compared with the groups without MSC infusion, but the effect was greater in the combined therapy group [262]
Exercise and stem cell transplantation	Effects of the combined treatment of bone marrow stromal cells with mild exercise and thyroid hormone on brain damage and apoptosis in a mouse focal cerebral ischemia model [279]	MSCs	Decrease in infarct volume and decrease in apoptosis [279]

## Abbreviations

**Abbreviation**	**Meaning**
tPA	tissue plasminogen activator
HSCs	hematopoietic stem cells
NSCs	neural stem cells
MSCs	mesenchymal stem cells
IPSCs	induced pluripotent stem cells
MAP2	microtubule-associated protein 2
ECM	extracellular matrix
GDNF	glial cell line-derived neurotrophic factor
EPCs	endothelial progenitor cells
USC-Exos	urine-derived stem cells
NPCs	neural progenitor cells
MMPs	matrix metalloproteinases
TBI	traumatic brain injury
SVZ	subventricular zone
IV	intravenous
OPCs	oligodendrocyte progenitor cells
BM-MSCs	bone marrow-derived mesenchymal stem cells
NVU	neurovascular unit
IGF-1	insulin-like growth factor
VEGF	vascular endothelial growth factor
EGF	epidermal growth factor
bFGF	basic fibroblast growth factor
BMPs	bone morphogenic proteins
MCAO	middle cerebral artery occlusion
SDF-1	stromal cell-derived factor-1
CXC4	chemokine receptor type 4
IL	interleukin
PGE2	prostaglandin E2
FGF2	fibroblast growth factor 2
VEGFR2	vascular endothelial growth factor receptor 2
BDNF	brain-derived neurotrophic factor
PD	Parkinson’s disease
SGZ	subgranular zone
BBB	blood-brain barrier
SGZ	subgranular zone
PDGF	platelet-derived growth factor
OGD/R	oxygen glucose deprivation/re-oxygenation
IC	intracerebral
IA	intra-arterial
TNF-α	tumor necrosis factor-α
DAMPs	damage-associated molecular patterns
MCP-1	monocyte chemoattractant protein-1
TGF-β	transforming growth factor-β
NGF	nerve growth factor
PEI	polyethyleneimine
CCR4	CC chemokine receptor 4
ROS	reactive oxygen species
I/R	ischemia/reperfusion
CUECD2	CUE domain-containing 2
NPs	nanoparticles
IFN-γ	interferon-γ
GFAP	glial fibrillary acidic protein
EVs	extracellular vesicles
UC-MSCs	umbilical cord-mesenchymal stem cells
HIF-1α	hypoxia-inducible factor-1α
CTZ	coelenterazine
TSPO	translocator protein
EE	enriched environment
VR	virtual reality
BCI	brain-computer interfaces
FES	functional electrical stimulation
TMS	transcranial magnetic stimulation
NTF	neurofeedback therapy
